# Comparison of Outcomes Between Two-Screw Proximal Femoral Nail and Halifax Femoral Nail in Elderly Patients With Intertrochanteric Fractures

**DOI:** 10.7759/cureus.103124

**Published:** 2026-02-06

**Authors:** Samay Jaiswal, Harshvardhan Buddhist, Himanshu Chaudhary, Ravikant Maurya, Sudhanshu, Ashish Ranjan

**Affiliations:** 1 Orthopedics and Traumatology, Institute of Medical Sciences, Banaras Hindu University, Varanasi, IND; 2 Orthopedics, Institute of Medical Sciences, Shri Ram Murti Smarak Hospital, Bareilly, IND; 3 Orthopedics and Trauma, Max Hospital, New Delhi, IND; 4 Orthopedics and Traumatology, Dr. KNS Memorial Institute of Medical Sciences, Barabanki, IND; 5 Orthopedics, Institute of Medical Sciences, Banaras Hindu University, Varanasi, IND

**Keywords:** complications, halifax femoral nail, outcomes, proximal femur nail, trochantric fracture, two-screw pfn

## Abstract

Introduction: Trochanteric fractures represent a substantial proportion of hip fractures in the elderly and are associated with significant morbidity and mortality. Dynamic hip screw fixation has shown higher failure rates in unstable fractures, leading to the use of intramedullary implants such as the proximal femoral nail (PFN) and the Halifax (Gamma) nail.

Methods: A cross-sectional study was conducted on 134 patients aged 60-80 years with unstable trochanteric fractures admitted to the Trauma Center of the Institute of Medical Sciences, Banaras Hindu University, Varanasi, Uttar Pradesh, India, from 2019 to 2021. Patients were randomly divided into two equal groups (n = 67 each), receiving either PFN (Nebula Surgical, Rajkot, India) or Halifax nail (GESCO Healthcare, Chennai, India) fixation. Clinical and radiological outcomes were assessed at one, three, six, and 12 months, and complications were recorded.

Results: The mean age of patients was 69.3 years, with a female predominance. The mean operative duration was significantly shorter in the Halifax group (45.9 minutes) compared to the PFN group. Patients with the Halifax nail achieved full weight-bearing earlier (eight weeks) and had fewer complications, such as the Z-effect, screw back-out, and femoral shaft fractures, which were observed more frequently in the PFN group. Union rates were similar in both groups (93.4%). The mean tip-apex distance (TAD) was lower in the Halifax group (14.4 ± 3.2 mm) compared to the PFN group (20.9 ± 2.7 mm).

Conclusion: The Halifax femoral nail demonstrated several advantages over the PFN, including shorter operative time, earlier mobilization, and fewer complications, making it a promising option for managing unstable trochanteric fractures in elderly patients.

## Introduction

Trochanteric fractures represent a significant cause of morbidity and mortality among the elderly, accounting for nearly 40% of all hip fractures [1]. These injuries typically result from low-energy trauma and extend from the extracapsular basilar region of the femoral neck to the area near the lesser trochanter, prior to the formation of the medullary canal [2].

The outcome of treatment for trochanteric fractures depends on several factors, including the patient's age, general health status, time elapsed since injury, quality of surgical management, and the stability achieved at fixation [3]. Contemporary surgical approaches primarily involve two major categories of implants: extramedullary devices such as the dynamic hip screw (DHS) and intramedullary devices, including the proximal femoral nail (PFN) and the Halifax (Gamma) nail [4].

Use of DHS has been associated with higher failure rates in unstable intertrochanteric fractures due to complications such as femoral neck collapse and limb shortening. Consequently, DHS is considered less suitable for unstable fracture patterns [4].

To address the limitations of plate-based fixation, Arbeitsgemeinschaft für Osteosynthesefragen (AO)/Association for the Study of Internal Fixation (ASIF) introduced the cephalomedullary PFN in 1996, designed for trochanteric entry [5]. This implant offers superior biomechanical stability compared to DHS and is associated with reduced intraoperative blood loss, lower infection rates, decreased morbidity, and earlier mobilization [6]. Furthermore, the PFN provides additional medial column support, promoting fracture healing even without complete anatomical restoration [7].

Another widely utilized intramedullary device for unstable trochanteric fractures is the Gamma or Halifax nail. The Halifax femoral nail incorporates a sliding lag screw mechanism that permits controlled compression across the fracture site. However, the Gamma nail design has a recognized structural weak point at the lag screw insertion hole, where the cross-sectional area is reduced by approximately 73% [8].

Given these considerations, the present study was undertaken to compare the short-term outcomes of trochanteric fractures treated surgically using PFN or Halifax femoral nail.

## Materials and methods

This prospective study was conducted at the Trauma Center of the Institute of Medical Sciences, Banaras Hindu University, Varanasi, Uttar Pradesh, India, from 2019 to 2021. A total of 134 patients aged 60-80 years suffering from intertrochanteric femur fractures were included after obtaining approval from the institutional ethical committee (approval no. 2956). These patients were selected and randomly divided into two treatment groups (n = 67 per group): those receiving the PFN (Nebula Surgical, Rajkot, India) and those receiving the Halifax femoral nail (GESCO Healthcare, Chennai, India). The fractures were classified according to Evans' classification based on X-ray findings [9], and unstable fractures were planned for inclusion in the study. After primary stabilization using skin traction in the preoperative period, patients were planned for surgery after all pre-anesthetic clearances, and those patients who were fit for surgery were enrolled in the study. Proper consent was obtained from all the patients. All surgeries were performed by the same surgeon using the same surgical technique.

Operative technique

Spinal or epidural anesthesia was administered to all patients according to the surgeon’s and anesthesiologist’s preferences. Patient positioning was the same for both the conventional two-screw PFN and Halifax nail. The patients were placed in the supine position on a fracture table, with the contralateral leg flexed and abducted to accommodate the image intensifier (Allengers Medical Systems, Chandigarh, India). The operative leg was put on traction, and the upper body was tilted to the unaffected side for comfortable insertion of the nail.

Closed reduction was achieved on the table and confirmed under the image intensifier. In some cases, Steinmann pins (Nebula Surgical) were used as a joystick to reduce the fracture percutaneously. Occasionally, mini-open methods were employed with bone spikes when anatomical reduction was not possible with closed reduction. The standard AO approach was used for both types of nailing. The tip of the greater trochanter was identified by palpation in thin patients and using the image intensifier in obese patients. A 5-cm longitudinal incision was made proximal to the tip of the greater trochanter, and then the gluteus medius muscle was split in line with the fibers. Entry was confirmed in both anteroposterior (AP) and lateral views with the help of an awl (Nebula Surgical). A guidewire (Nebula Surgical) was inserted, and proximal reaming was done using a 15-mm proximal reamer (Nebula Surgical). Reaming of the canal was performed in a serial fashion, starting with eight reamers (Nebula Surgical) and finishing when there was sufficient resistance in the femoral canal. The nail was then manually inserted over the guidewire into the femoral canal using twisting movements of the hand under C-arm guidance until the hole for the screw (Nebula Surgical) was at the level of the inferior margin of the neck. Light blows with a hammer may be carefully applied if needed.

In the conventional PFN group, an additional guidewire (Nebula Surgical) was placed through the sleeve and jig (Nebula Surgical) for the derotation screw (Nebula Surgical) placement. After satisfaction, drilling was performed up to the tip of the guidewire, and the appropriately sized screw was inserted. Closure was performed in layers, and a sterile dressing was applied.

All patients in both groups were allowed toe-touch weight-bearing immediately in the postoperative period with a walker and received the same postoperative rehabilitation. These patients were followed up at one, three, six, and 12 months postoperatively and then yearly thereafter. Clinical and radiological variables, as well as postoperative complications, were compared between the two groups. In subsequent follow-ups, patients were advised to undergo X-ray imaging and were assessed for union and complications. Union was defined as signs of new bone growth, i.e., callus formation that completely bridges the fracture site. Increased weight-bearing was determined based on the amount of callus present on serial X-rays and the patient’s clinical tolerance for weight-bearing in both groups. The X-rays were also assessed for complications such as varus collapse, screw cut-out, broken nail, Z-effect, and non-union.

Patients younger than 60 years, with pathological fractures, a history of previous surgery on the proximal femur, old non-unions and malunions, trochanteric fractures older than one week, and patients unfit for surgery were excluded from the study.

Statistical analysis

Data were collected and analyzed using IBM SPSS Statistics version 20.0 software (IBM Corp., Armonk, USA). Categorical data were evaluated by calculating frequency and percentages, and then compared using chi-square tests. For quantitative data, means and standard deviations were calculated and compared using t-tests.

## Results

A statistically significant difference (p-value < 0.05) was observed between the two study groups with respect to the time of surgery, time of full weight-bearing, neck-shaft angle, and tip-apex distance (TAD) at one-year follow-up. A statistically insignificant difference (p-value > 0.05) was observed between both genders.

The study revealed that most patients were in the age group of 60-75 years, with a mean age of 69.3 years. Female patients were more affected than male patients (11:9). The surgery duration was 30-50 minutes in most patients, with full weight-bearing achieved in around eight weeks. In both groups, most patients were not subjected to any secondary procedures.

**Figure 1:**
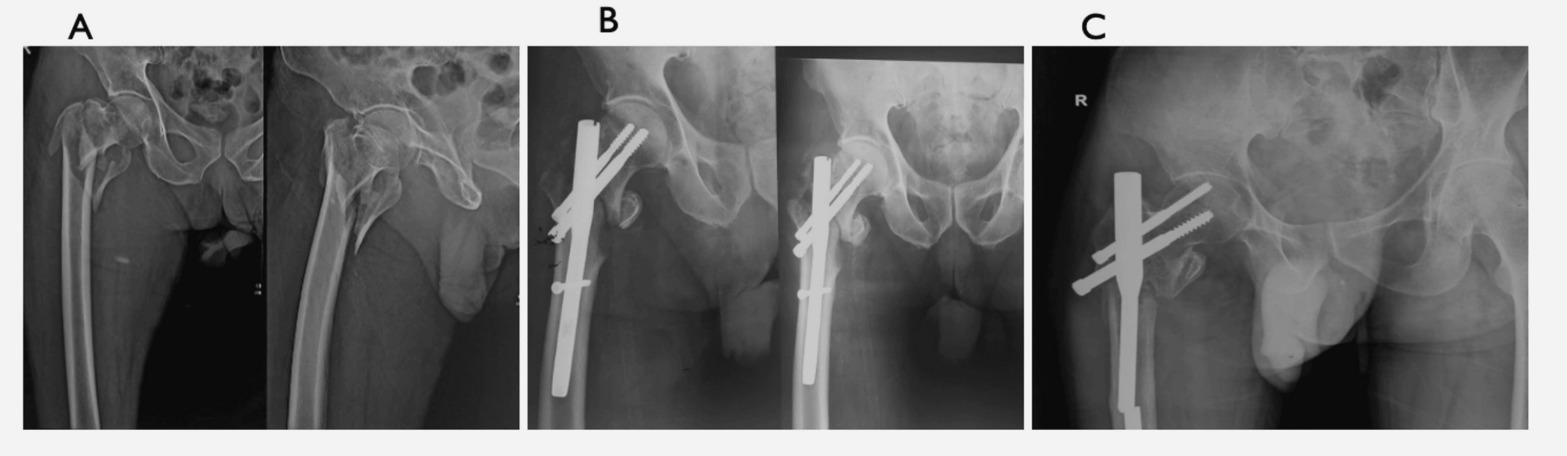
Broken PFN at follow-up (A) Preoperative intertrochanteric femur fracture. (B) Two-month follow-up after surgery. (C) Four-month follow-up showing a broken PFN. PFN: proximal femur nail

In the present study, complications at the end of one year were assessed in both groups. Among patients treated with PFN, varus collapse was observed in four cases (6.7%), femoral shaft fracture in two cases (3.3%), non-union in four cases (6.7%), broken nail in two cases (3.3%), and Z-effect in two cases (3.3%) (Figure 1).

**Figure 2:**
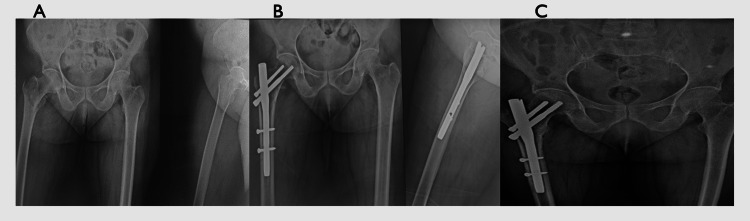
Fracture completely united at six months using a PFN (A) Preoperative intertrochanteric femur fracture. (B) Two-month follow-up after surgery. (C) Six-month follow-up after surgery showing a united fracture. PFN: proximal femur nail

**Figure 3:**
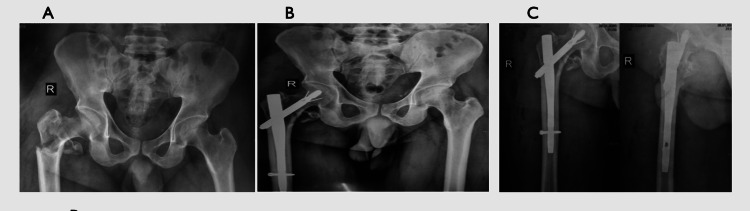
Fracture completely united at six months using a Halifax nail (A) Preoperative intertrochanteric femur fracture. (B) Two-month follow-up after surgery. (C) Six-month follow-up after Halifax nail fixation showing a united fracture.

In contrast, the Halifax group had fewer complications, with varus collapse and non-union occurring in two cases each (3.3%). The follow-up X-rays showing union for both the conventional PFN and Halifax nail are depicted in Figures 2-3, respectively.

Notably, no cases of femoral shaft fracture, broken nail, or Z-effect were observed in the Halifax group. Screw cut-out was not reported in either group. The complications were assessed and compared in both groups (Table 1).

**Table 1:** Comparison of complications between the PFN and Halifax nail groups at one year PFN: proximal femur nail

Complcation	PFN group		Halifax nail group	
	N	%	N	%
Varus collapse	4	6.70%	2	3.30%
Femoral shaft fracture	2	3.30%	0	0.00%
Non-union	4	6.70%	2	3.30%
Screw cut-out	0	0.00%	0	0.00%
Broken nail	2	3.30%	0	0.00%
Z-effect	2	3.30%	0	0.00%
Distal lock	67	100.00%	63	93.30%
Total	67	100%	67	100%

When comparing functional and radiological outcomes between the two groups at one year, the mean age was comparable (69.6 ± 7.7 years in PFN vs. 69.3 ± 9.6 years in Halifax; p = 0.871). The mean duration of surgery was significantly longer in the PFN group (80.4 ± 10.5 minutes) compared to the Halifax group (45.9 ± 8.5 minutes; p < 0.001). Patients in the PFN group required a longer time to achieve full weight-bearing (9.8 ± 1.6 weeks) compared to those in the Halifax group (8.1 ± 2.4 weeks; p = 0.001). The mean hospital stay was slightly longer in the PFN group (5.6 ± 1.6 days) than in the Halifax group (4.7 ± 3.2 days), although this difference was not statistically significant (p = 0.206).

The functional outcome measured by the Harris Hip Score [10] was similar in both groups (84.3 ± 7.6 in PFN vs. 83.7 ± 5.9 in Halifax; p = 0.735). Radiologically, the mean TAD was significantly greater in the PFN group (20.9 ± 2.7 mm) compared to the Halifax group (14.4 ± 3.2 mm; p < 0.001). The mean neck-shaft angle was slightly lower in the PFN group (128.1 ± 2.6°) than in the Halifax group (130.5 ± 3.1°; p = 0.002). Fracture healing time was nearly similar in both groups (12.0 ± 1.5 weeks in PFN vs. 12.2 ± 1.8 weeks in Halifax; p = 0.564). Activities of daily living (ADL) scores were also comparable (2.7 ± 0.8 in PFN vs. 2.8 ± 0.6 in Halifax; p = 0.580) (Table 2).

**Table 2:** Comparison of different parameters between the PFN and Halifax nail groups at one year * denotes statistical significance (p ≤ 0.05). TAD: tip-apex distance; ADL: activities of daily living; df: degree of freedom; PFN: proximal femur nail

Parameter	PFN group		Halifax nail group		t-value	df	p-value
								Mean	SD	Mean	SD
	Age (years)	69.6	7.7	69.3				9.6	0.163	58	0.871
Time of surgery (minutes)	80.4	10.5	45.9	8.5	13.986	58	<0.001*
Time of full weight-bearing (weeks)	9.8	1.6	8.1	2.4	3.336	58	0.001*
Hospital stay (days)	5.6	1.6	4.7	3.2	1.279	58	0.206
Hip Harris Score	84.3	7.6	83.7	5.9	0.34	58	0.735
TAD (mm)	20.9	2.7	14.4	3.2	8.519	58	<0.001*
Neck shaft angle (°)	128.1	2.6	130.5	3.1	-3.183	58	0.002*
Fracture healing time (weeks)	12	1.5	12.2	1.8	-0.58	56	0.564
ADL	2.7	0.8	2.8	0.6	-0.557	58	0.580

A comparison of parameters between both genders is depicted in Table 3.

**Table 3:** Comparison of parameters in both genders at one year TAD: tip-apex distance; ADL: activities of daily living; df: degree of freedom

Parameter	Male		Female		t-value	df	p-value
	Mean	SD	Mean	SD			
Age (years)	68.9	10.4	69.9	7.0	-0.423	58	0.674
Time of surgery (minutes)	62.7	20.7	63.5	19.3	-0.138	58	0.891
Time of full weight-bearing (weeks)	9.3	2.1	8.7	2.2	1.061	58	0.293
Hospital stay (days)	5.8	3.4	4.6	1.4	1.875	58	0.066
Hip Harris Score	83.8	6.4	84.2	7.2	-0.190	58	0.850
TAD (mm)	16.9	3.9	18.2	4.8	-1.184	58	0.241
Neck shaft angle (°)	128.7	3.3	129.8	2.8	-1.487	58	0.143
Fracture healing time (weeks)	12.2	1.7	12.0	1.6	0.285	56	0.776
ADL	2.9	0.7	2.7	0.6	1.459	58	0.150

## Discussion

Unstable intertrochanteric fractures are seen more frequently in older adults, particularly those with reduced bone mineral density [11]. These fractures typically involve substantial comminution and demonstrate a greater tendency for displacement following fixation. The PFN was developed by integrating the advantages of an unreamed intramedullary femoral nail with a sliding, load-sharing femoral neck screw. This combination enhances rotational control of the proximal fragment. Additionally, the nail tip was specially engineered to minimize stress concentration, thereby lowering the risk of femoral shaft fractures during or after surgery [12].

Both the Halifax nail and PFN provide biomechanically robust fixation that facilitates early mobilization and is associated with relatively low complication rates. The PFN offers axial and rotational stability due to its dual-screw configuration [13]. In this design, the antirotation screw stabilizes the proximal fragment, while the lag screw enables controlled compression across the fracture site.

In our study, the average patient age was 69.3 years, aligning closely with findings of Kuderna et al. [14], who reported a mean age of 68 years, ranging from 21 to 94 years. The mean operative time for Halifax nail fixation in our cohort was 45.9 minutes, comparable to the results of Sharma et al. [15], who documented an average operating time of 40 minutes. In contrast, the mean operative time for PFN in our study was 80.4 minutes, which differs from the observations of Mohan et al. [16], who reported an average duration of 50 minutes for proximal femoral nail antirotation (PFNA) procedures.

The Harris Hip Score [10], a widely accepted, joint-specific functional assessment tool, was used in our study to evaluate postoperative outcomes at one, three, six, and 12 months. Kashid et al. [17] similarly utilized the Harris Hip Score to compare PFN and Gamma nail fixation, reporting no significant differences between the two groups - a finding that parallels our study results.

Sharma et al. [18] concluded that Gamma nails demonstrated superior outcomes in the treatment of trochanteric fractures. Consistent with their findings, our study also demonstrated favorable results with Halifax nail fixation, achieved using a minimally invasive technique that provides reliable stabilization across a variety of fracture patterns.

Multiple studies have reported postoperative hip and thigh pain as common complications following intramedullary fixation [12,19]. In our study, 90.1% of patients reported such discomfort during follow-up, although this did not significantly affect postoperative function. These symptoms may be attributable to the intramedullary approach itself. McConnell et al. [20] demonstrated that insertion of an intramedullary device predictably results in injury to the gluteus medius tendon, which could explain postoperative pain in the hip and thigh.

McConnell et al. [20] also described typical mobilization timelines, beginning with toe-touch weight-bearing from postoperative day two, followed by full weight-bearing at approximately 6-8 weeks. More than 60% of patients achieved unassisted full weight-bearing by 8-9 weeks, while 18% did so between 12 and 14 weeks - differences largely influenced by age and bone quality. Our findings were comparable. We initiated toe-touch weight-bearing from the first postoperative day and advanced weight-bearing gradually based on radiographic evidence of callus formation. Notably, patients treated with the Halifax nail achieved full weight-bearing earlier.

Fracture stability was assessed based on union of the posteromedial cortex, correction of obliquity, and quality of reduction. Union occurred in 93.4% of cases in both the PFN and Halifax groups, consistent with findings reported by Singh et al. [21].

Common complications leading to fixation failure include varus collapse and Z-effect, often attributed to poor reduction or osteoporotic bone. The Halifax nail design did not predispose to the Z-effect, which was observed in two PFN cases. The presence of the tri-wire system in the Halifax nail enhances anchorage in osteoporotic bone and reduces lag screw back-out, suggesting a mechanical advantage over the PFN. Additionally, nail breakage and femoral shaft fractures have been documented with PFN but are rare with Halifax nails. These complications generally arise when patients prematurely bear full weight before adequate fracture healing; none were observed in the Halifax cohort in our study.

TAD exceeding 25 mm is a recognized risk factor for varus collapse. In our study, mean TAD values were 20.9 ± 2.7 mm for PFN and 14.4 ± 3.2 mm for Halifax nails, both within acceptable limits.

The typical duration of hospitalization was 3-5 days. Longer stays, extending up to 21 days, occurred in patients with associated injuries or uncontrolled comorbidities. For example, the individual with the longest stay had additional chest trauma and a distal humerus fracture. Some delays were also related to COVID-19-associated logistical challenges. In two Halifax cases, distal locking required additional effort due to an instrumentation mismatch between the jig and the distal locking holes.

Overall, our study demonstrated that complications such as Z-effect, screw back-out, and femoral shaft fractures - frequently associated with PFN - were not encountered with the Halifax nail. Union rates were consistently high, operative duration was shorter, and full weight-bearing was achieved earlier in the case of the Halifax implant. Considering its favorable complication profile, shorter operative time, and earlier rehabilitation, the Halifax femoral nail appears to be a more efficient and promising option for the management of intertrochanteric fractures in elderly patients.

## Conclusions

With fewer complications, fast operating times, and early load-bearing, the Halifax femoral nail is a better and more promising treatment option for managing intertrochanteric fractures in elderly patients. We observed that the operative time was significantly shorter and full weight-bearing could be started earlier with the Halifax nail compared to PFN. Complications such as Z-effects, screw back-out, and femoral shaft fractures were not observed with the Halifax nail, whereas they were common with PFN, suggesting higher union rates with the Halifax nail. Considering the above results in patients with osteoporosis and complex trochanteric fractures, the Gamma or Halifax nail with additional stabilizing tri-wire can be the treatment of choice.
